# Addressing Adolescent Depression in Tanzania: Positive Primary Care Workforce Outcomes Using a Training Cascade Model

**DOI:** 10.1155/2017/9109086

**Published:** 2017-11-26

**Authors:** Stan Kutcher, Yifeng Wei, Heather Gilberds, Adena Brown, Omary Ubuguyu, Tasiana Njau, Norman Sabuni, Ayoub Magimba, Kevin Perkins

**Affiliations:** ^1^Dalhousie University and the IWK Health Centre, 5850 University Avenue, P.O. Box 9700, Halifax, NS, Canada B3K 6R8; ^2^Sun Life Financial Chair in Adolescent Mental Health, Dalhousie University and IWK Health Centre, Halifax, NS, Canada; ^3^Sun Life Financial Chair in Adolescent Mental Health Team, IWK Health Centre, Halifax, NS, Canada; ^4^Farm Radio International, Ottawa, ON, Canada; ^5^Muhimbili National Hospital, Kalenga Street, P.O. Box 65000, Dar es Salaam, Tanzania; ^6^Muhimbili University of Health and Allied Sciences, P.O. Box 65001, Dar es Salaam, Tanzania; ^7^Mental Health and Substance Abuse, Ministry of Health, P.O. Box 9083, Dar es Salaam, Tanzania; ^8^Non-Communicable Disease, Ministry of Health, P.O. Box 9083, Dar es Salaam, Tanzania

## Abstract

**Background:**

This is a report on the outcomes of a training program for community clinic healthcare providers in identification, diagnosis, and treatment of adolescent Depression in Tanzania using a training cascade model.

**Methods:**

Lead trainers adapted a Canadian certified adolescent Depression program for use in Tanzania to train clinic healthcare providers in the identification, diagnosis, and treatment of Depression in young people. As part of this training program, the knowledge, attitudes, and a number of other outcomes pertaining to healthcare providers and healthcare practice were assessed.

**Results:**

The program significantly, substantially, and sustainably improved provider knowledge and confidence. Further, healthcare providers' personal help-seeking efficacy also significantly increased as well as the clinicians' reported number of adolescent patients identified, diagnosed, and treated for Depression.

**Conclusion:**

To our knowledge, this is the first study reporting positive outcomes of a training program addressing adolescent Depression in Tanzanian community clinics. These results suggest that the application of this training cascade approach may be a feasible model for developing the capacity of healthcare providers to address youth Depression in a low-income, low-resource setting.

## 1. Introduction

Depression is one of the most common mental disorders globally, with a substantial lifetime prevalence rate [1, 2]. It is projected to become the leading cause of disability worldwide by the year 2030 and is currently the leading cause of disability among those aged 15 to 44 [3, 4]. Africa carries the largest burden of disease of any continent and has significant challenges in the provision of mental healthcare [5–7]. Atilola [8] proposes that a majority of the global burden of mental disorders will be in sub-Saharan Africa (SSA) by the year 2020.

Adolescence is a critical period of development that is characterized by numerous health challenges [9] including the time of the highest onset of mental disorders, such as Depression, of which about 50% of cases can be diagnosed prior to 25 years of age [3, 4, 10–13]. The average global prevalence rate of Depression among those aged 5 to 17 is approximately 6%; however, there is little data available regarding the prevalence of Depression in this age group in sub-Saharan Africa [14]. The data that does exist is sparse and varies widely from country to country, from 8.6% in Uganda, 10.7%–21.1% in Tanzania (for pregnant young mothers), to 41% in South Africa [15–17].

In Tanzania, about 70% of the population consists of those aged between 0 and 24 years [18]. Due to gradually increasing life-expectancy, this cohort will mature into adulthood, bringing with them a high prevalence of Depression resulting in increased pressures for mental healthcare systems that are unable to address current needs. For this reason, there is a pressing need to address the issue of adolescent Depression in Tanzania and other sub-Saharan countries as soon as possible and, given the magnitude of the challenge, it should be addressed at the primary healthcare level [19, 20].

Currently, a majority of Tanzania's population relies on traditional/alternative medicine and mental illness is the second most common condition managed by practitioners of traditional medicine [21]. Alternatively mental disorders are often treated by primary healthcare providers, such as community health workers, general nurses, and community health officers [22–25], who have received very limited, if any, training in the delivery of mental healthcare to adolescents. At this time there exists no community based healthcare providers in Tanzania who have been trained in the clinical capacities needed to identify, diagnose, and treat Depression in young people.

Tanzania, one of the world's poorest countries, has one of the lowest physician to population ratios: 1.4 healthcare providers (HCPs) per 1000 individuals [26]. Further, there are 0.04 psychiatrists and 0.005 psychologists per 100,000 population, and an overall total of 0.3 mental health workers (including psychologists, psychiatrists, nurses, and other mental health providers) per 100,000 population [23, 27–29]. Primary healthcare is generally provided through district hospitals, community health centres, or dispensaries [30]. Failure to recognize mental disorders as a priority in health policy and funding, stigmatization of patients, and specialty mental healthcare providers, poor mental health literacy, and a lack of mental health competencies among community based healthcare providers are all additional factors associated with challenges in delivery of mental healthcare to young people [31–34].

To effectively address the challenge of adolescent Depression in Tanzania, we applied an approach delivered as a Grand Challenges Canada funded project called “An Integrated Approach to Addressing the Challenge of Depression among the Youth in Malawi and Tanzania” (IACD). This approach consists of three components that taken together create an integrated pathway through care for young people with Depression. These are as follows: stigma reduction through youth focused radio programs; mental health literacy training applied through schools; and health provider workforce capacity development for the identification, diagnosis, and treatment of adolescent Depression in community health centres (http://teenmentalhealth.org/new-initiatives-posts/addressing-adolescent-depression-challenge/).

## 2. Methods

### 2.1. Study Design

In a sample of community based healthcare providers (HCPs), we conducted a longitudinal cohort study and measured participant knowledge, attitudes (stigma), clinical care confidence, and personal mental health help-seeking efficacy at specific time points: pretraining (at the beginning of the first training period); posttraining (at the conclusion of the first training session); prerefresher (about four months following the completion of the first training session); postrefresher (at the conclusion of the refresher training session); and postrefresher follow-up (about four months following the refresher training session).

### 2.2. Participants

Participants were recruited from the Arusha and Meru regions in Tanzania with the support of the Tanzanian Ministry of Health and under direction of the District Health Authorities. Most government healthcare facilities were invited to participate. Two nurse officers who held the position of mental health coordinators (one in Arusha and one in Meru) assisted in the recruitment of HCPs. Training session participants included HCPs working in community healthcare settings as well as in community based mental healthcare units. Most training session participants had at least five years of clinical experience but the majority had never treated any psychiatric patients. Some trainees were nurses who had received a general adult focused mental healthcare exposure in their preservice courses. No participant had previously received training in the diagnosis and treatment of adolescents with mental illness, including Depression.

Participants completed an anonymous survey at baseline (*n* = 61), immediately following the initial training (*n* = 59), at the beginning of the refresher training (*n* = 48), immediately after the refresher training (*n* = 49), and once during a follow-up period after the refresher training (*n* = 51). Participants consisted of clinicians working in the participating community clinics and consisted of clinical officers; senior clinical officers; nurses (registered nurses and midwives); and other community healthcare providers (HCPs).

### 2.3. Procedures

With support of the Ministry of Health (NS and AM) and the assistance of a number of nationally recognized mental health experts (including OU and TN), we first adapted an adolescent Depression education program developed for use in Canada by one of the authors (SK) and certified by the Canadian College of Family Physicians. From this adaptation, SK created a training program that was then modified and contextualized for use in Tanzania by OU and TN. Using a training cascade model [28, 29, 31, 35–37] four master trainers with extensive mental health experience were trained by SK. They then trained a larger group of lead trainers who then trained available healthcare providers working in community clinics located in the Districts of Meru and Arusha as chosen by the project team. Lead trainers were supported during their work by an electronic program under the management of OU and TN. Initial training to HCPs was delivered over a period of five consecutive days and then a refresher training intervention of four consecutive days was provided approximately four months later.

The initial training provided a general overview of adolescent development and other mental disorders commonly occurring in young people. It then addressed clinical competencies for the identification, diagnosis, and treatment of adolescent Depression including the use of diagnostic checklists, severity, and outcome measures and treatment guidelines. Participants were further taught a psychotherapeutic intervention (Effective Helping, EH) based on counseling and cognitive behavior therapy that was developed specifically to meet needs of youth within the African context. Pharmacotherapy training addressed the appropriate use of fluoxetine as the medication of choice delivered concurrently with EH or secondarily if the EH intervention alone had not led to positive results. Fluoxetine, an appropriate medication treatment for adolescent Depression [38, 39], replaced the commonly prescribed antidepressant medication amitriptyline, a compound not appropriate for treating Depression in young people [40, 41].

Participants received similar content during the refresher training but the training focused more on content areas that participants felt they needed further training based on their clinical experience following completion of the initial training program. Approximately four months following the end of the refresher intervention, a final data collection occurred.

### 2.4. Outcomes

Outcomes were assessed with an “in-class” pen and paper evaluation using a questionnaire that includes six sections: Section  A contains 30 questions designed to assess HCPs knowledge about the identification, diagnosis, and treatment of Depression in young people (Cronbach's *α* pretest = 0.756). For each question, participants were instructed to respond “True,” “False,” or “Do Not Know” by marking an** X** in the appropriate box. If participants selected “Do Not Know,” selected more than one option (without a clear indication of one option being crossed off), or did not select any option, the corresponding question was marked as incorrect. Correct answers received a score of 1; all other answers including incorrect answers or “Do Not Know” answers received a score of 0. Participants could receive a potential lowest score of 0 with a potential highest score of 30. Section  B requested HCPs to rate their confidence levels regarding the diagnosis and treatment of Depression on a four item 4-point Likert scale (Cronbach's *α* pretest = 0.880). Scores ranged from not confident (1), somewhat confident (2), very confident (3), to extremely confident (4) with a potential highest score of 16. Section  C asked about HCPs' healthcare practices related to adolescent Depression. HCPs reported how many adolescent patients they diagnosed and treated for Depression compared to the three months prior to their training if any. Response options included 0, 1–5, 6–9, and 10+. Participants were also asked to indicate, of the people they treated for Depression, what percent would they put in the categories of very much worse, much worse, worse, no change, improved, much improved, and very much improved, based on the Clinical Global Impressions (CGI) tool [42]. Section  D asked questions related to the following: (1) HCPs anxiety when talking to or treating a person with a mental health problem; (2) HCPs attitudes towards people with a mental health problem; (3) the likelihood of HCPs suggesting a friend or a family member to get help for a mental health problem; and (4) the likelihood of HCPs to get help for themselves for a mental health problem if they thought help was needed. Section  E asked three questions addressing HCPs mental health help-seeking efficacy as a result of participation in the training programs. Participants were asked to answer either “Yes” or “No” if they had suggested that a friend or family member or a colleague or coworker seeks help for a mental health problem or disorder or if they themselves sought help for a mental health problem or disorder. If they said “No” they were also asked if they thought such help was needed. The same questionnaire was administered at all time points except that Sections  C to E were not asked during initial training, and Sections  D and E were not asked during the refresher training follow-up. The questionnaires used for this study have been provided in Supplementary Material available online at https://doi.org/10.1155/2017/9109086.

The evaluation of the training program was designed to answer the following questions:

1. Does the training on adolescent Depression, using the cascade train-the-trainer approach, increase knowledge of adolescent Depression among frontline health workers in Tanzania and do any observed knowledge benefits from the training persist over time?
2. Does the training on adolescent Depression, using the train-the-trainer approach, increase self-reported confidence in dealing with adolescent Depression among frontline health workers in Tanzania and if so, does this persist over time?
3. Does the training on adolescent Depression have an effect on self-reported healthcare practices (the number of patients being identified, diagnosed, or treated for Depression) by frontline health workers in Tanzania?
4. Does the training on adolescent Depression improve HCPs' attitudes (decrease stigma) related to mental disorders?
5. Does the training on adolescent Depression increase self-reported help-seeking behavior for a mental health concern among frontline health workers in Tanzania?

### 2.5. Analysis

Analyses were performed using Friedman's test; post hoc analyses were conducted using Wilcoxon signed-rank tests with a Bonferroni correction applied, resulting in a significance level set at* p* ≤ 0.01. Using the Kolmogorov-Smirnov (K-S) test, the knowledge scores at initial pretraining, *D*(61) = 0.106,* p* = 0.086, did not deviate significantly from normal; however, knowledge scores at initial posttraining, *D*(59) = 0.141,* p* = 0.005, refresher pretraining, *D*(48) = 0.125,* p* = 0.056, refresher posttraining, *D*(49) = 0.158,* p* = 0.004, and refresher follow-up, *D*(51) = 0.374,* p* < 0.000, were significantly nonnormal. All analyses were completed using SPSS Statistics software for Windows, Version 22.0. Due to the small sample of follow-up assessments from the initial training session (*n* = 13), these data were excluded from analysis.

## 3. Results

### 3.1. Participants

Participants were both males and females and included all the different healthcare provider designations found in community health clinics in Tanzania. Details are provided in Table 1. In relation to the professional categories, “clinicians” included clinical officers and senior clinical officers; “nurses” included registered nurses and midwives; and “other” included medical attendants and other health professionals. During the initial evaluations, participants were not asked to provide demographic information on their age or gender; the details provided in Table 1 are from the refresher evaluations (*N* = 48).

### 3.2. Knowledge

**Figure 1:**
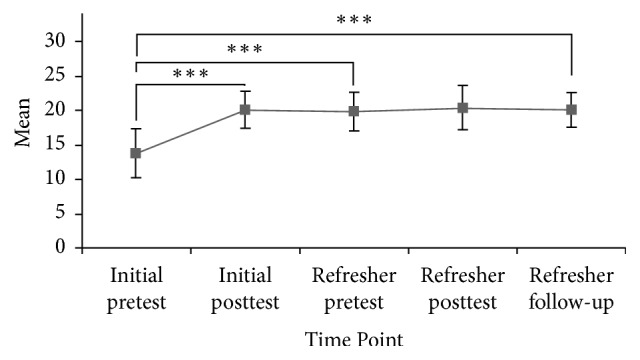
Knowledge scores of HCP over time. Error bars represent ±1 SD. ^*∗∗∗*^*p* ≤ 0.001.

Knowledge scores were significantly and substantially positively affected as a result of the initial training [*F*(4) = 76.711,* p* < 0.001]. Mean knowledge scores between initial pre- (*M* = 13.84, SD ± 3.55, *n* = 61) and posttraining (*M* = 20.14, SD ± 2.70, *n* = 59) improved significantly; *Z* = 6.322,* p* ≤ 0.001; *r* = 0.82. Mean knowledge scores between initial pre (*M* = 13.84, SD ± 3.55, *n* = 61) and refresher pre (*M* = 19.90, SD ± 2.80, *n* = 48) continued to demonstrate significant improvement *Z* = 5.798,* p* ≤ 0.001; *r* = 0.84. Mean knowledge scores between initial pre (*M* = 13.84, SD ± 3.55, *n* = 61) and refresher follow-up (*M* = 20.12, SD ± 2.53, *n* = 51) continued to show significant improvement; *Z* = 5.736,* p* ≤ 0.001; *r* = 0.80. At all points, effect sizes meet or exceed conditions for a large effect (*r* = 0.80); see Figure 1.

### 3.3. Self-Rated Confidence

**Figure 2:**
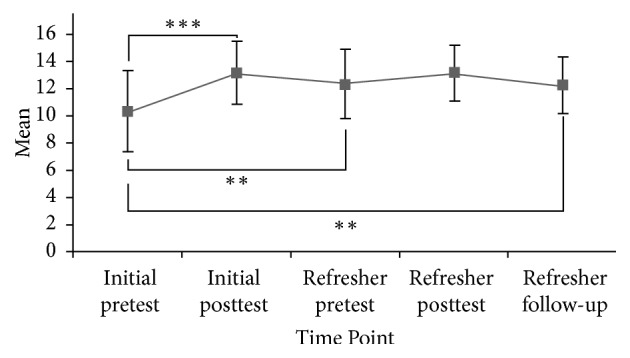
Confidence scores of HCP over time. Error bars represent ±1 SD. ^*∗∗*^*p* ≤ 0.005. ^*∗∗∗*^*p* ≤ 0.001.

Confidence scores were significantly and positively affected as a result of the initial training [*F*(4) = 27.792,* p* < 0.001]. Mean confidence scores between initial pre- (*M* = 10.28, SD ± 2.96, *n* = 57) and posttraining (*M* = 13.14, SD ± 2.33) showed a statistically significant improvement; *Z* = 4.644,* p* ≤ 0.001; *r* = 0.61. Mean confidence scores between initial pre (*M* = 10.28, SD ± 2.96, *n* = 57) and refresher pre (*M* = 12.31, SD ± 2.54, *n* = 48) continued to demonstrate significant improvement; *Z* = 2.800,* p* ≤ 0.005; *r* = 0.40. Mean confidence scores between initial pre (*M* = 10.28, SD ± 2.96, *n* = 57) and refresher follow-up (*M* = 12.22, SD ± 2.10, *n* = 50) also continued to show significant improvement; *Z* = 2.886,* p* ≤ 0.005; *r* = 0.41. At all points, effect sizes approach or exceed conditions for a moderate effect (*r* = 0.50); see Figure 2.

### 3.4. Healthcare Provision

**Figure 3:**
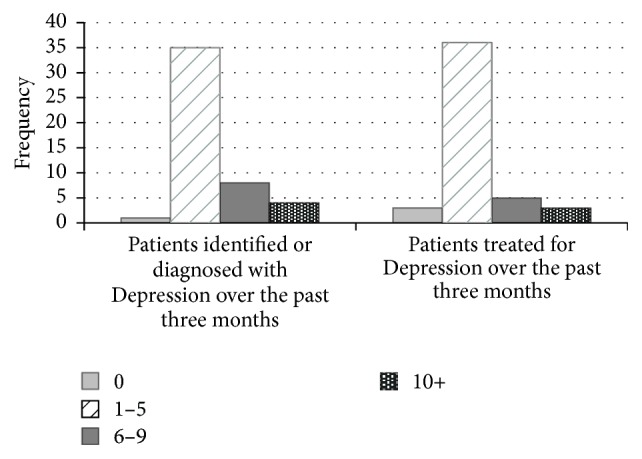
Healthcare practices of HCP after training.

At the onset of the refresher training, participants were asked to indicate how many patients they had identified or diagnosed with Depression in the past three months as a result of the training compared to the three months prior to their initial training session. Of the available choices, 2.1% reported no change; 72.9% reported between 1 and 5 youths; 16.7% reported between 6 and 9 youths, and 8.3% reported over 10 youths (see Figure 3). Therefore, the number of adolescent patients identified or diagnosed with Depression in the interval between the first and refresher training sessions compared to a similar time prior to the first training session was in the range of a minimum of 123 to more than 287 among the 48 HCPs reporting.

At the onset of the refresher training, participants were also asked to indicate how many more patients they had treated for Depression in the past three months as a result of the training than in the three months prior to their initial training. Of the available choices, 6.4% reported no difference; 76.6% reported between 1 and 5 youths; 10.6% reported between 6 and 9 youths; and 6.4% reported over 10 youths (see Figure 3). Therefore, the number of patients treated for Depression was in the range of a minimum of 96 to more than 255 among the 47 HCPs reporting.

### 3.5. Improvement in Depression with Treatment

At the onset of the refresher (*n* = 30) and at follow-up to the refresher (*n* = 34) training, participants were asked to provide an overall change score (based on the Clinical Global Improvement Scale: CGI) of youth that they had treated for Depression. The mean CGI score prerefresher was 4.70 (SD ± 0.70) and the mean CGI score at refresher follow-up was 5.06 (SD ± 0.78). These mean scores translate to improved and much improved, respectfully. Mean CGI scores between prerefresher (*M* = 4.70, SD ± 0.70) and refresher follow-up (*M* = 5.06, SD ± 0.78) did not show a statistically significant change in treatment outcomes *Z* = −1.746,* p* ≥ 0.05; *r* = 0.32, which remained overall improved/much improved.

### 3.6. Thoughts and Feelings

At the onset of the refresher, training participants were asked about their own perceptions and actions related to their initial training and the subsequent period of their clinical application of that training. With respect to self-reported anxiety about half (48%) of the participants reported that they felt more anxious about talking to or treating a person with a mental illness while almost half (43%) reported feeling less anxious. In terms of stigma against people with mental illness, all participants indicated that their attitudes towards people with mental illnesses or mental health problems had improved.

When asked about the likelihood of suggesting that others obtain help for a mental health concern as a result of what they had learned in their training session, 98% of HCPs indicated that they were more likely to suggest that a friend or family member gets help for a mental disorder or mental health problem. When asked about the likelihood of getting help for themselves, 2% of HCPs felt about the same and 98% indicated that they were more likely to get help for a mental health problem if they thought they needed to.

### 3.7. Help-Seeking Behavior

**Figure 4:**
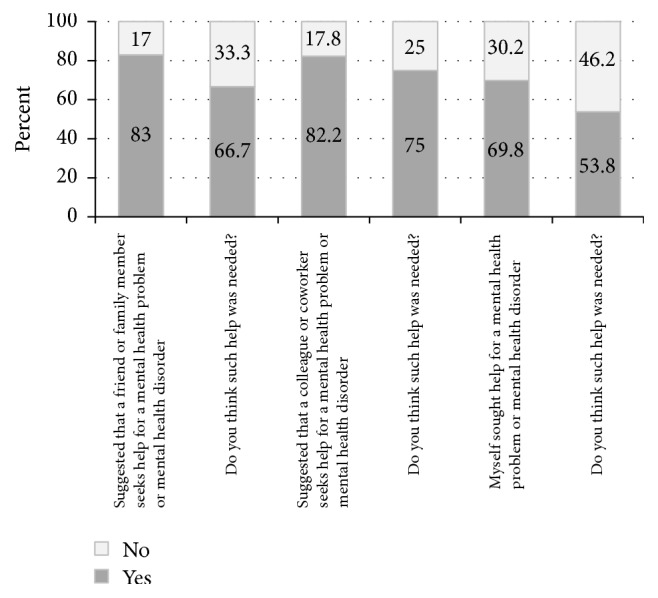
Help-seeking among HCPs.

At the onset of the refresher training, participants were asked about their help-seeking behavior in the past three months as a result of the initial training they received (see Figure 4). When asked whether HCPs suggested that a friend or family member seeks help for a mental health problem or disorder, 39 (83%) HCPs indicated that they had and 8 (17%) had not. When asked whether HCPs suggested that a colleague or coworker seeks help for a mental health problem or disorder, 37 (82.2%), as a result of the training that they had received, HCPs indicated they had and 8 (17.8%) had not. When asked whether HCPs themselves sought help for a mental health problem or disorder as a result of the training that they had received, 30 (69.8%) indicated they had and 13 (30.2%) had not.

## 4. Discussion

The results of this intervention demonstrate significant, substantial, and sustained positive impact of training in the diagnosis and treatment of Depression in adolescents provided to a representative group of community clinic based healthcare professionals in Tanzania, using a cascade training model. Specifically, training significantly improved knowledge, decreased stigma, and enhanced provider self-confidence in this clinically important area. The format of the refresher training (using a participant driven question and answer approach) proved to be a useful method for addressing issues that community clinic health providers had encountered in their clinical work following their initial training exposure but did not demonstrate a booster effect on further significantly increasing knowledge or confidence. These results suggest not only that was the training program effective using this cascade model but that the results were self-sustaining over time after a single implementation of the training program. However, this was not a randomized controlled study and, thus, the results must remain speculative. Further research is warranted to determine whether this type of cascade training model could be rolled out in other districts, as an effective way to reach large numbers of community clinic health providers across the country of Tanzania. With these cautions in mind, this study, still, has contributed to identifying a positive step in helping improve mental health literacy, particularly as it relates to adolescent Depression, in a low-resource setting.

Although indirectly, we were able to demonstrate the impact of this training cascade model on clinical care delivered in community clinics. As reported by HCPs involved in this intervention, within the three-month period prior to the training program initiation, there were no cases of adolescents diagnosed or treated for Depression in any of the participating clinics. In the three months following the training, HCPs estimated that they had diagnosed between 123 and 287 adolescents and treated between 96 and 255 adolescents for Depression demonstrating the practical “on-the-ground” effect of this training program on actual healthcare provision and practice of HCPs. For a one-year period, this total could be extrapolated to be at least 246 patients diagnosed and 192 treated from a sample of 48 participants or an average of 5 adolescents diagnosed and 4 adolescents treated for Depression per HCP per year. Should this training program be able to reach and train large numbers of HCPs and if the current provider calculation of impact proves to be correct at the country level, the potential scale-up of this cascade training approach may be able to reach thousands of young people with Depression who heretofore have not been diagnosed or treated. However, we are aware that the estimations of identified and treated youth will need to be verified by clinical records and not just based on clinician recall. Thus, this possibility, although promising, must remain speculative at this time. Further research is now underway to address this issue.

Additionally, HCPs reported that overall their patients were considered to be “improved” or “much improved” as a result of the treatment provided at the two time points that this outcome was evaluated. While this evaluation was based on the CGI scale that the HCPs had been trained to use, in the absence of individual discrete patient data based on objective measures, this finding must be interpreted with caution. HCPs used both the Effective Helping psychological intervention taught in the training sessions and fluoxetine which was made available by the Ministry of Health for use as the first-line pharmacologic intervention in treatment of adolescent with Depression, replacing the previously available tricyclic antidepressants (TCAs) which are not recommended for use in this population. HCPs were taught when and how to properly apply fluoxetine in their training program. However, because a clinical audit was not conducted as part of this intervention, we are not able to determine how appropriately the clinical protocol related to fluoxetine use was followed. Further study of this issue would provide valuable insights into actual practice.

Of interest is that the training participants were roughly equally divided in reporting if they felt more or less anxious when talking to a person with a mental health problem or mental illness as a result of the training. This may suggest that the lack of prior training in mental healthcare, particularly in adolescent Depression, led to some HCPs initially feeling anxious when interacting with young patients who may have mental health related problems, when they previously had not done so. On the other hand, it may suggest that the training experience provided some HCPs with more insight about mental disorders in young people, particularly Depression, leading some to feel less anxious when interacting with patients who may have mental health related problems or a mental disorder.

All of the HCPs indicated their attitudes towards people with a mental illness improved, indicating the training program was effective in increasing HCPs' attitudes (decreasing stigma) towards people with a mental disorder. This was an important outcome of the training experience given the significant impact that stigma against people with mental disorders has in sub-Saharan Africa [43–49]. If replicated in other applications of this training program across the country, this simple intervention could have a major impact on decreasing stigma and concurrently increasing access to mental healthcare for those young people who require it.

Another impact of the training experience was on the HCPs own self-reported help-seeking efficacy, in terms of both help-seeking intent and help-seeking behaviors compared to before the training exposure. After the training, almost all of HCPs (98%) indicated they were at least more likely to suggest that a friend or family member goes and gets help and that they themselves would be more likely to seek help for a mental illness or mental health problem. When asked about their actual help-seeking, 83% reported that since the training program they had suggested that a friend or family member seeks help, 82.2% had suggested that a colleague or coworker seeks help and 69.8% of HCPs indicated they had sought help for themselves. Thus, the training program not only demonstrated positive effects among the targeted population, adolescents, but also, indirectly, positively impacted the behaviors of the HCPs participating in the training. Thus, there may be an additional “value-add” to this intervention, in that the training program may be reaching HCPs as well as their patients. This suggests the training program may also be effective in addressing workforce mental healthcare needs and may sensitize HCPs to act positively towards addressing the mental healthcare needs that they and their friends and family members have. If these individuals (HCPs and their personal networks) do seek help for their mental health, further improvements may be achieved among the larger population, simply by training healthcare providers in the identification, diagnosis, and treatment of youth Depression. Such a value-added component has, to our knowledge, not been reported in any other studies of mental health training for African healthcare providers.

Should this cascade approach be replicated outside of the two target regions of this study, this may provide a useful and inexpensive way of reaching the widely dispersed community clinic health provider workforce across Tanzania. This approach could thereby enhance access to mental healthcare for young people with Depression. If this training can be built upon to incorporate other common mental disorders arising in young people, it may be a means of creating widespread capacity to address the mental healthcare needs of a large segment of the population that is currently not being served. Such an approach can feasibly be applied in both urban and rural areas but will require a coordinated implementation process under the leadership of the Ministry of Health. At the date of this writing, although there has been a strong interest from the Ministry in application of this approach and a mental healthcare policy review in which this intervention is being considered as a component is underway, scale-up has not yet begun. Before a scale-up should be implemented, further evaluation of this training cascade model and training program may be useful. As part of the next stop of the IACD project, an intervention is currently underway in a number of health clinics to better establish the clinical outcomes of patients interacting with HCPs who have received this intervention.

## 5. Limitations

This study is of a cohort of community healthcare providers followed over a period of one year and not a controlled study. Thus, in the absence of a controlled study, we cannot fully conclude that the outcomes reported herein were solely the result of the intervention described. However, while an alternative explanation is possible, that is, not highly probable as no other interventions addressing these issues were underway concurrently and the participants were not exposed to any other mental health-training programs that may have influenced their activities. While a controlled study of actual clinical practice would be ideal, such research is uncommon, even in high-income countries and may not be easily applied in this or similar low-income settings. Thus, replication of this intervention in another sub-Saharan country, if providing similar results, would give greater comfort to the validity of these findings. A further limitation was our use of self-report to evaluate stigma and help seeking. Similarly we did not compare self-reports of help seeking with actual clinic data but given the challenges of routine clinic data collection such sophisticated cross-reference may not be easily applied. How to better study actual help-seeking behaviors will need to be considered for future work. We did not use a validated stigma measure applied in a pre-post-follow-up design and future research should also consider that type of more robust evaluation.

In relation to using self-report, another issue that arose is that some sections of the assessment form were not completed correctly. For instance, for Section  A, knowledge, participants sometimes provided more than one answer per question. In such cases, these responses were marked as “incorrect.” Furthermore, in Section  C, when asked to indicate their perception of change in Depression status of those they had treated for Depression in percent, many respondents simply checked one response. As a result we treated this question as a 6-point Likert scale ranging from very much worse (0) to very much improved (6). For participants that did provide percent, the category with the highest percent provided was the one used for analysis.

Additionally, as mentioned, the response rate for participant recruitment was between 70 and 80 percent of those invited to participate. The two nurse officer coordinators, tasked with recruiting HCPs from outpatient facilities for inclusion in the study, indicated that some HCPs were not able to participate in the mental health-training program due to limited availability of human resources. In facilities outside of the district and regional hospitals (dispensaries, community healthcare centres, etc.), there are very limited resources, including numbers of HCPs. In these settings if one HCP is absent for an extended period of time, this can place an undue burden on those left behind. This is a challenge that will need to be solved at the system level in order to be able to build needed capacity, particularly in rural and remote areas.

## 6. Conclusion

In summary, this is, to our knowledge, the first report of the positive impact of a training and refresher training cascade approach to addressing the challenge of adolescent Depression in Tanzania. It has demonstrated a positive impact, not only on knowledge, attitudes, confidence, and personal help-seeking for community based healthcare providers, but also on enhancing access to community based mental healthcare and treatment outcomes for young people with Depression. While additional research including randomized controlled trials is needed to more confidently be able to evaluate clinical outcomes, this approach, if replicated in other settings, has the potential to be scaled-out not only across Tanzania but also potentially across sub-Saharan Africa.

## Supplementary Material

Outcomes Evaluation Questionnaire.

## Figures and Tables

**Table 1:** Demographic characteristics of healthcare providers.

Variable	*N*	Percentage (%)
Gender (*n* = 46)		
Male	20	43.5
Female	26	56.5
Professional category (*n* = 47)		
Clinician	31	66.0
Nurse	8	17.0
Other	8	17.0

	Range	Mean ± SD

Age (*n* = 48)	25–59	39.90 ± 9.95
