# Supplementary material for: Addressing Adolescent Depression in Tanzania: Positive Primary Care Workforce Outcomes Using a Training Cascade Model
Source: Depress Res Treat. 2017 Nov 26;2017:9109086. doi: 10.1155/2017/9109086 (PMC5733241; doi:10.1155/2017/9109086)
Supplement: Supplementary file 1 — Outcomes Evaluation Questionnaire. [file 9109086.f1.zip › Suppl.docx]

**Appendix**

**Knowledge Assessment for Trainers and Clinical Staff: Tanzania**

The following 30 questions have been designed to assess your knowledge about the identification, diagnosis and treatment of Depression in young people. Please put in a response to every question by putting an “X” in the appropriate box, either: True; False; Don’t Know. If you do not know the answer please use the “Don’t Know” box instead of guessing.

Please complete all of these questions. They will be used to link the responses you have made on this evaluation to response you have made on other evaluations. This allows for analysis of group (not an individual’s) difference in responses over time. Please do not put your name on this evaluation as all materials are confidential.

**Professional Designation:** ____________________________ **Location:** __________________________

**Today’s** **Date:** ____________________________ **Favorite colour:** __________________________

**Favorite animal:** ________________________ **Favorite number:** __________________________

**Section A:** For each of the following statements select **True, False,** or **Do Not Know** by marking an **X** in the appropriate box.

| **Question** | **True** | **False** | **Do Not Know** |
| --- | --- | --- | --- |
| 1. It is normal for teenagers to be depressed much of the time |  |  |  |
| 1. Many mental disorders begin during the teenage years |  |  |  |
| 1. The diagnosis of Depression in a teenager is usually made using a blood test |  |  |  |
| 1. The diagnosis of Depression in a teenager requires the presence of a persistent depressed or irritable mood plus at least five other specified symptoms for a period of at least two weeks |  |  |  |
| 1. The best way to establish therapeutic trust with a teenager is to know about teen music |  |  |  |
| 1. The prevalence of Depression by the end of the teenage years is about six to eight percent of the population |  |  |  |
| 1. Usual life stresses such as having an argument with parents or doing poorly at school often lead to Depression in a teenager |  |  |  |
| 1. The KADS-6 is a useful tool to help identify Depression in a teenager |  |  |  |
| 1. Psychological and medication treatments for Depression in teenagers usually do not work very well |  |  |  |
| 1. EBM Depression is a psychological treatment that has been shown to help treat adolescents with Depression |  |  |  |
| 1. The following are ALL symptoms of Depression in a teenager: fatigue; concentration problems; depressed mood; loss of pleasure |  |  |  |
| 1. One useful medication for treating Depression in a teenager is Amytriptyline |  |  |  |
| 1. Depressed teenagers should be encouraged to watch TV or work on their computers at night, to help cheer them up |  |  |  |
| 1. Depressed teenagers who are feeling tired should be advised to avoid strenuous exercise so as to help conserve their energy |  |  |  |
| 1. Depressed teenagers should be encouraged to drink plenty of caffeine and sugar containing drinks to help them get more energy |  |  |  |
| 1. The TeFa is a useful clinical tool to help evaluate the day to day functioning of a teenager with depression |  |  |  |
| 1. The CGI is a helpful test to identify a teenager who has psychotic symptoms plus Depression |  |  |  |
| 1. A family history of Depression is a significant risk factor for Depression in a teenager |  |  |  |
| 1. Depression in a teenager is an important suicide risk factor |  |  |  |
| 1. Because suicide in teenagers is so rare, busy health care providers should not routinely do an assessment of suicide risk when diagnosing Depression in a teenager |  |  |  |
| 1. The TASR-Am is a good way of predicting which teenager is likely to die by suicide |  |  |  |
| 1. Because they are so tired and unhappy, depressed teenagers should never use alcohol |  |  |  |
| 1. The CRAFFT is a useful tool to help a clinician identify a teenager at risk for problems with alcohol or drug use |  |  |  |
| 1. A clinician should not be concerned with keeping confidentiality with a teenager because s/he is not yet an adult |  |  |  |
| 1. The KADS-11 should be used to BOTH help with the diagnosis of Depression and to measure the severity of Depression in a teenager |  |  |  |
| 1. If using Fluoxetine to treat Depression in a teenager, the target daily dose for treatment is 20mg per day continued for a period of 10 weeks |  |  |  |
| 1. TCYH is a self-help health and mental health diary that can be used by a teenager with Depression during treatment |  |  |  |
| 1. Whenever possible, an alternative treatment should be used instead of a standard treatment, especially if it is more appealing to the Depressed teenager or his/her parents |  |  |  |
| 1. During the first ten weeks of treatment the Depressed teenager should be seen weekly in the clinic |  |  |  |
| 1. The teenagers' family should rarely if ever be involved in his/her treatment |  |  |  |

**Section B:** For the next four statements, please put an “**X**” in the box that most closely identifies how you think or feel about that statement. Please put your response in for every statement.

| **Question** | **Not Confident** | **Somewhat Confident** | **Very Confident** | **Extremely Confident** |
| --- | --- | --- | --- | --- |
| 1. I feel confident in my knowledge about Depression in teenagers |  |  |  |  |
| 1. I feel confident in being able to effectively provide treatment to a teenager with Depression |  |  |  |  |
| 1. I feel confident in being able to talk to young people and their parents about Depression |  |  |  |  |
| 1. I feel confident in being able to talk to other health care providers about Depression in teenagers |  |  |  |  |

**Retention Assessment for Trainers and Clinical Staff**

The following questions have been designed to assess your knowledge about the identification, diagnosis and treatment of Depression in young people. Please respond to every question by putting an **“X”** in the appropriate box, either: True; False; Don’t Know. If you do not know the answer please use the “Don’t Know” box instead of guessing.

Please complete all of these questions. They will be used to link the responses you have made on this evaluation to response you have made on other evaluations. This allows for analysis of group (not an individual’s) difference in responses over time. Please do not put your name on this evaluation as all materials are confidential.

**Profession: Health Facility:** **Date:**

**Age: Sex: Favorite animal:**

**Please check this box if you are a TOT**

**Section A:** For each of the following select “True”, “False” or “I Don’t Know” by marking an **“X”** in the appropriate box.

|  | **Questions** | **True** | **False** | **I Don’t Know** |
| --- | --- | --- | --- | --- |
|  | It is normal for teenagers to be depressed much of the time |  |  |  |
|  | Many mental disorders begin during the teenage years |  |  |  |
|  | The diagnosis of Depression in a teenager is usually made using a blood test |  |  |  |
|  | The diagnosis of Depression in a teenager requires the presence of a persistent depressed or irritable mood plus at least five other specified symptoms for a period of at least two weeks |  |  |  |
|  | The best way to establish therapeutic trust with a teenager is to know about teen music |  |  |  |
|  | The prevalence of Depression by the end of the teenage years is about six to eight percent of the population |  |  |  |
|  | Usual life stresses such as having an argument with parents or doing poorly at school often lead to Depression in a teenager |  |  |  |
|  | The KADS-6 is a useful tool to help identify Depression in a teenager |  |  |  |
|  | Psychological and medication treatments for Depression in teenagers usually do not work very well |  |  |  |
|  | EBM Depression is a psychological treatment that has been shown to help treat adolescents with Depression |  |  |  |
|  | The following are ALL symptoms of Depression in a teenager: fatigue; concentration problems; depressed mood; loss of pleasure |  |  |  |
|  | One useful medication for treating Depression in a teenager is Amytriptyline |  |  |  |
|  | Depressed teenagers should be encouraged to watch TV or work on their computers at night, to help cheer them up |  |  |  |
|  | Depressed teenagers who are feeling tired should be advised to avoid strenuous exercise so as to help conserve their energy |  |  |  |
|  | Depressed teenagers should be encouraged to drink plenty of caffeine and sugar containing drinks to help them get more energy |  |  |  |
|  | The TeFa is a useful clinical tool to help evaluate the day to day functioning of a teenager with depression |  |  |  |
|  | The CGI is a helpful test to identify a teenager who has psychotic symptoms plus Depression |  |  |  |
|  | A family history of Depression is a significant risk factor for Depression in a teenager |  |  |  |
|  | Depression in a teenager is an important suicide risk factor |  |  |  |
|  | Because suicide in teenagers is so rare, busy health care providers should not routinely do an assessment of suicide risk when diagnosing Depression in a teenager |  |  |  |
|  | The TASR-Am is a good way of predicting which teenager is likely to die by suicide |  |  |  |
|  | Because they are so tired and unhappy, depressed teenagers should never use alcohol |  |  |  |
|  | The CRAFFT is a useful tool to help a clinician identify a teenager at risk for problems with alcohol or drug use |  |  |  |
|  | A clinician should not be concerned with keeping confidentiality with a teenager because s/he is not yet an adult |  |  |  |
|  | The KADS-11 should be used to BOTH help with the diagnosis of Depression and to measure the severity of Depression in a teenager |  |  |  |
|  | If using Fluoxetine to treat Depression in a teenager, the target daily dose for treatment is 20mg per day continued for a period of 10 weeks |  |  |  |
|  | TCYH is a self-help health and mental health diary that can be used by a teenager with Depression during treatment |  |  |  |
|  | Whenever possible, an alternative treatment should be used instead of a standard treatment, especially if it is more appealing to the Depressed teenager or his/her parents |  |  |  |
|  | During the first ten weeks of treatment the Depressed teenager should be seen weekly in the clinic |  |  |  |
|  | The teenagers' family should rarely if ever be involved in his/her treatment |  |  |  |

**Section B:** For the next four statements, please put an “X” in the box that most closely identifies how you think or feel about that statement. Please complete all four statements.

| **Question** | **Not Confident** | **Somewhat Confident** | **Very Confident** | **Extremely Confident** |
| --- | --- | --- | --- | --- |
| 1. I feel confident in my knowledge about Depression in teenagers |  |  |  |  |
| 1. I feel confident in being able to effectively provide treatment to a teenager with Depression |  |  |  |  |
| 1. I feel confident in being able to talk to young people and their parents about Depression |  |  |  |  |
| 1. I feel confident in being able to talk to other health care providers about Depression in teenagers |  |  |  |  |

**Section C:** The following questions are about your health care practice since participating in the training program.

1. Over the past three months how many more patients have you **identified** or **diagnosed** with Depression compared with the three months prior to your training?

0 1 – 5 6 – 9 10+

1. Over the past three months how many more patients have you **treated** for Depression compared with the three months prior to your training?

0 1 – 5 6 – 9 10+

1. Of the people you have treated for Depression what percent would you put in the following categories?

Very Much Improved _____%

Much Improved _____%

Improved _____%

No Change _____%

Worse _____%

Much Worse _____%

Very Much Worse _____%

**Section D:** Please answer these following questions comparing how you think or feel **NOW** compared to **BEFORE** you attended the mental health training program

1. I feel ______ when talking to or treating a person with a mental illness or mental health problem:

Much less anxious ____; less anxious ____; about the same ____; more anxious ____; much more anxious ____.

1. My attitude towards a person with a mental illness or mental health problem is:

Much improved ____; improved ____; about the same ____; negative ____; much more negative ____.

1. I am ______ to suggest that a family member or friend get help for a mental disorder or mental health problem:

Much more likely ____; more likely ____; the same ____; less likely ____; much less likely ____.

1. I am ____ to get help for a mental disorder or mental health problem if I think I need to:

Much more likely ____; more likely ____; the same ____; less likely ____; much less likely ____.

**Section E:** In the last three months, AND BECAUSE OF WHAT I HAVE LEARNED ABOUT MENTAL HEALTH AND MENTAL DISORDERS IN THIS PROGRAM I have:

Suggested that a friend or family member seek help for a mental health problem or mental disorder: Yes ___; No ___. If No, do you think such help was needed: Yes ___; No ___

Suggested that a colleague or co-worker seek help for a mental health problem or mental disorder. Yes ___; No ___. If No, do you think such help was needed: Yes ___; No ___

Myself sought help for a mental health problem or mental disorder. Yes ___; No ___. If No, do you think such help was needed: Yes ___; No ___

**THANK YOU FOR YOUR PARTICIPATION IN THIS SURVEY. ALL ANSSWERS TO ALL THE QUESTIONS WILL BE KEPT CONFIDENTIAL.**
